# Vitamin C revisited

**DOI:** 10.1186/s13054-014-0460-x

**Published:** 2014-08-06

**Authors:** Heleen M Oudemans-van Straaten, Angelique ME Spoelstra-de Man, Monique C de Waard

**Affiliations:** Department of Intensive Care, VU University Medical Centre, De Boelelaan 1117, Amsterdam, 1081 HZ the Netherlands

## Abstract

This narrative review summarizes the role of vitamin C in mitigating oxidative injury-induced microcirculatory impairment and associated organ failure in ischemia/reperfusion or sepsis. Preclinical studies show that high-dose vitamin C can prevent or restore microcirculatory flow impairment by inhibiting activation of nicotinamide adenine dinucleotide phosphate-oxidase and inducible nitric oxide synthase, augmenting tetrahydrobiopterin, preventing uncoupling of oxidative phosphorylation, and decreasing the formation of superoxide and peroxynitrite, and by directly scavenging superoxide. Vitamin C can additionally restore vascular responsiveness to vasoconstrictors, preserve endothelial barrier by maintaining cyclic guanylate phosphatase and occludin phosphorylation and preventing apoptosis. Finally, high-dose vitamin C can augment antibacterial defense. These protective effects against overwhelming oxidative stress due to ischemia/reperfusion, sepsis or burn seems to mitigate organ injury and dysfunction, and promote recovery after cardiac revascularization and in critically ill patients, in the latter partially in combination with other antioxidants. Of note, several questions remain to be solved, including optimal dose, timing and combination of vitamin C with other antioxidants. The combination obviously offers a synergistic effect and seems reasonable during sustained critical illness. High-dose vitamin C, however, provides a cheap, strong and multifaceted antioxidant, especially robust for resuscitation of the circulation. Vitamin C given as early as possible after the injurious event, or before if feasible, seems most effective. The latter could be considered at the start of cardiac surgery, organ transplant or major gastrointestinal surgery. Preoperative supplementation should consider the inhibiting effect of vitamin C on ischemic preconditioning. In critically ill patients, future research should focus on the use of short-term high-dose intravenous vitamin C as a resuscitation drug, to intervene as early as possible in the oxidant cascade in order to optimize macrocirculation and microcirculation and limit cellular injury.

## Introduction

Critically ill patients suffer from multiple organ dysfunction mostly occurring in the course of ischemia/reperfusion or septic shock. In these conditions, overwhelming amounts of reactive oxygen species (ROS) and reactive nitrogen species are generated. ROS are oxidizing agents produced during mitochondrial respiration and phagocytosis. In addition, ROS cause post-translational modifications of proteins, modifying their action and affecting cellular signaling, gene expression, oxygen sensing and other physiological processes [1,2]. In low concentrations, ROS also enhance the antioxidant response via nuclear factor-erythroid 2-related factor 2 activation, and thereby promote cell survival [2]. While ROS are crucial for body homeostasis and defense, they cause harm if abundant production overwhelms the antioxidant defense. In that case, ROS can induce reversible or irreversible injury to proteins, lipids and nucleic acids, thereby contributing to endothelial dysfunction, cellular injury and multiple organ dysfunction.

Endothelial dysfunction is a uniform, ROS-mediated manifestation of ischemia/reperfusion and sepsis. Furthermore, ROS-induced damage to the glycocalyx, cellular membranes and junctions leads to increased permeability, adhesion of leukocytes and platelets with local activation of inflammation and coagulation, leads to loss of endothelial vasodilatation and attenuates the vascular response to vasoconstrictors [3-6]. Subsequent hypotension, vascular leakage and microcirculatory flow impairment at reperfusion augment tissue hypoxia due to increased diffusion distance for oxygen and may thereby enhance cellular damage and organ failure [7,8]. Ascorbate, the redox form of vitamin C, is a physiological antioxidant. We hypothesize that the early administration of a high pharmacological dose of vitamin C to patients with sepsis or after ischemia/reperfusion can reduce oxidative damage to endothelial and other cells, and thereby improve tissue perfusion and oxygenation, and mitigate subsequent organ dysfunction [9,10].

Vitamin C also has other effects. Vitamin C improves immune function, and facilitates enteral uptake of nonheme iron, reduction of folic acid intermediates and synthesis of collagen (wound healing), cortisol, catecholamines and carnitine [11-13]. These effects are beyond the scope of this review.

The aim of this narrative review is to summarize the role of vitamin C in mitigating ROS-induced damage to endothelial and myocardial cells in ischemia/reperfusion or sepsis. By limiting endothelial dysfunction, vitamin C might improve tissue perfusion and reduce tissue hypoxia and subsequent organ dysfunction. Experimental and clinical studies on the use of vitamin C are reported with a focus on cardiovascular effects.

## Review

### Pathophysiology

**Figure 1:**
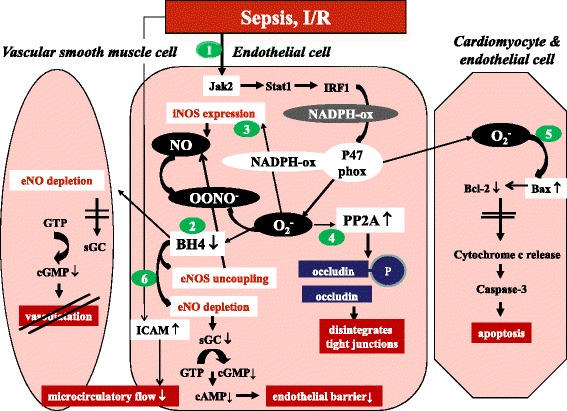
**Ischemia/** **reperfusion-induced and sepsis-induced endothelial dysfunction is initiated by increased amounts of reactive oxygen species.** 1. Ascorbate reduces the production of superoxide (O_2_^–^), hydrogen peroxide and peroxynitrite (OONO^–^) by inhibiting the Jak2/Stat1/IRF1 signaling pathway, which leads to subunit p47phox expression of nicotinamide adenine dinucleotide phosphate oxidase (NADPH-ox) and thus to O_2_^–^ formation. 2. Ascorbate protects against oxidative stress induced pathological vasoconstriction and loss of endothelial barrier by inhibiting tetrahydrobiopterin (BH_4_) oxidation, the cofactor of endothelial nitric oxide synthase (eNOS), thereby preventing endothelial nitric oxide (eNO) depletion and eNOS uncoupling. 3. Ascorbate inhibits inducible nitric oxide synthase (iNOS) mRNA and iNOS expression, preventing abundant production of nitric oxide (NO) that generates OONO^–^ in the presence of O_2_^–^. 4. Ascorbate protects against vascular leakage by inhibiting protein phosphatase 2A (PP2A) activation, which dephosphorylates occludin. Phosphorylated occludin is crucial for maintenance of tight junctions. 5. Ascorbate inhibits myocardial apoptosis by preventing Bax activation, which decreases the ability of BCl-2 to inhibit cytochrome-C release from the mitochondria into the cytoplasm and subsequent caspase-3 activation, which initiates apoptosis. The combination with vitamin E is synergistic. 6. Ascorbate inhibits microcirculatory flow impairment by inhibiting tumor necrosis factor-induced intracellular adhesion molecule (ICAM) expression, which triggers leukocyte stickiness and sludging. cAMP, cyclic adenosine monophosphate; cGMP, cyclic guanosine monophosphate; GTP, guanosine triphosphate; I/R, ischemia/reperfusion; sGC, soluble guanylate cyclase.

Ischemia/reperfusion-induced and sepsis-induced endothelial dysfunction is initiated by increased amounts of ROS produced by the induction of enzymes such as nicotinamide adenine dinucleotide phosphate-oxidase (NOX) and uncoupling of mitochondrial oxidative phosphorylation and endothelial nitric oxide synthase (eNOS). ROS are additionally produced by xanthine oxidase, lipoxygenase and cyclooxygenase, and during oxidation of catecholamines (Figure 1) [1,13].

Unopposed ROS oxidize tetrahydrobiopterin (BH_4_), the cofactor of eNOS, and thereby reduce eNOS activity, the enzyme producing endothelial nitric oxide (eNO). eNO initiates vasodilatation by stimulating soluble guanylcyclase and increasing cyclic guanosine monophosphate in smooth muscle cells [14]. It also inhibits platelet aggregation and adhesion of activated platelets and leukocytes. eNO is therefore crucial for patency of the microcirculation and its depletion hampers organ perfusion and oxygenation. In the absence of BH_4_, eNOS becomes uncoupled, producing superoxide (O_2_^–^) rather than nitric oxide (NO) [15]. O_2_^–^ and NO yield peroxynitrite, the most damaging ROS.

## Role of vitamin C: *in vitro* studies

The underlying mechanisms for the effect of ascorbate on these pathways have been demonstrated in *in vitro* studies with cultured endothelial cells. Endothelial cells can accumulate ascorbate to millimolar levels [16] and represent an appropriate model to study the effects of high-dose vitamin C. *In vitro* studies are presented focusing on protective effects of vitamin C (ascorbate) in ischemia/reperfusion and sepsis.

### Endothelial dysfunction

Ascorbate decreases oxidative stress in endothelial cells by reducing the production of O_2_^–^, hydrogen peroxide and peroxynitrite. Mechanisms include prevention of NOX activation, decreased inducible nitric oxide synthase (iNOS) expression and increased NO bioavailability (Figure 1) [16-18]. NOX is the major source of ROS in endothelial and myocardial cells [1,19]. Activation of NOX leads to the formation of intracellular O_2_^–^. Addition of ascorbate to endothelial cells exposed to oxidative stress prevented activation of NOX by inhibition of subunit p47phox expression (mediated by the Jak2/Stat1/IRF1 signaling pathway) [20]. NOX-derived ROS additionally increase the expression of iNOS [17], producing excessive NO. Abundant NO in the presence of O_2_^–^ generates peroxynitrite. Ascorbate prevented iNOS expression [16]. Ascorbate also scavenges O_2_^–^, but only at high levels ≥10 mmol/l [21].

Furthermore, ascorbate can increase NO bioavailability by preventing BH_4_ oxidation and recovering oxidized BH_4_ [18]. Recovery of BH_4_ by ascorbate prevents uncoupling of eNOS and associated O_2_^–^ production and restores eNOS activity and subsequent generation of eNO, which has a pivotal role in endothelial-dependent vasodilatation.

### Endothelial permeability

ROS additionally increase endothelial permeability [22], causing edema and contributing to organ dysfunction. Ascorbate can tighten the endothelial barrier through several pathways.

Constitutive eNO is required to control endothelial permeability and prevent loss of tight junctions between cells [23]. Loading endothelial cells with ascorbate preserved eNO generation through eNOS and decreased endothelial permeability. This effect depended on eNOS and guanylatecyclase, suggesting that tightening of the endothelium involved NO generation by eNOS and subsequent NO-dependent activation of guanylatecyclase [23].

Exposure of endothelial cells to lipopolysaccharide (LPS) increases endothelial permeability by inducing NOX-dependent protein phosphatase 2A activity and subsequent occludin dephosphorylation. Phosphorylated occludin is crucial to maintain tight junctions. Ascorbate protects against vascular leakage by inhibiting protein phosphatase 2A activation [24].

Furthermore, oxidants and LPS increase apoptosis, impairing the endothelial barrier. LPS decreases Bcl-2 (which inhibits apoptosis) and increases Bax (which suppresses the ability of Bcl2 to block apoptosis). Ascorbate inhibited apoptosis [25] and protected endothelial progenitor cells [26]. Simultaneous administration with vitamin E had a synergistic effect on the prevention of apoptosis.

### Impairment of microcirculatory flow

Oxidative stress stimulates the expression of tissue factors and cellular adhesion molecules at the surface of platelets and endothelial cells [27], promoting adhesion of leucocytes to the endothelium and formation of microthrombi and thus impairing microcirculatory flow. In cultured endothelial cells, ascorbate inhibited the tumor necrosis factor alpha-induced expression of intracellular adhesion molecule-1 in a dose-dependent manner [28], probably by modulating the production of ROS and reactive nitrogen species. By preventing intracellular adhesion molecule expression, ascorbate reduces leukocyte plugging in microvessels and microcirculatory flow impairment.

### Myocardial effects of vitamin C in ischemia/reperfusion

Ischemia/reperfusion injures not only the endothelium but also the myocardium, leading to stunning and arrhythmias. Loading isolated cardiomyocytes subjected to hypoxia/reoxygenation with ascorbate improved their resistance to cell death by decreasing ROS generation and inhibiting (proapoptotic) Bax expression, caspase-3 activation, and cytochrome-c translocation into the cytoplasm [29]. Pretreatment with vitamin C or vitamin E of isolated cardiomyocytes exposed to singlet oxygen reduced the number of hypercontracted cardiomyocytes in a concentration-dependent manner. Simultaneous administration of both vitamins acted synergistically [30].

### Immune effects of vitamin C in sepsis

In sepsis, ascorbate also influences macrophage activity and bacterial growth. Macrophages play an important role in sepsis, enhancing cytokine production as well as production of several types of ROS. ROS are necessary to overcome infections, but are only beneficial if their production is controlled. Incubation of macrophages with ascorbate regulated the phagocytic process by reducing adherence, chemotaxis, ingestion and O_2_^–^ anion production [31]. Furthermore, ascorbate has profound bacteriostatic activity. Ascorbate (in concentrations from 100 to 1,000 μM) significantly inhibited bacterial replication in dilute fecal samples *in vitro* [32].

## Role of vitamin C: animal studies

### Ischemia/reperfusion

Beneficial effects of ascorbate pretreatment on organ function were observed in ischemia reperfusion injury models of rat heart [33] and rabbit kidney [34], and of rat skeletal muscle [35,36], lung [37] and liver [38-41]. Studies are summarized in Table 1.

**Table 1:** **Pathophysiological effects and mechanisms of vitamin C in sepsis and ischemia reperfusion: animal studies**

**Model; dose and timing of ascorbate [study]**	**Pathophysiological effect**	**Mechanisms**
**Ischemia reperfusion**		
Cardiac arrest (VF-ES) in rats; 50 and 100 mg/kg i.v. at start of CPR [33]	Increases successful resuscitation after cardiac arrest rates and 72-hour survival (100 mg/kg better than 50 mg/kg)	Preservation of histology
		Reduced mitochondrial swelling
		Preserves mitochondrial respiration (complex I and IV)
		Inhibits MDA ↑
LAD coronary artery ischemia ± ischemic preconditioning in pigs; 2 g i.v. + 25 mg/minute before IPC or before ischemia [42]	Does not affect infarct size	
	Attenuates the beneficial effect of ischemic preconditioning indicating free oxygen radicals are involved in ischemic preconditioning	
Middle cerebral artery clamping in mice; DHA 40, 250 and 500 mg/kg, AA 250 and 500 mg/kg, before, 15 minutes and 3 hours after [81]	DHA gives dose-dependent:	DHA passes blood–brain barrier, ascorbate does not
	● Reperfusion blood flow ↑	No beneficial effect of ascorbate
	● Infarct size ↓	
	● Neurological deficit ↓	
	● Mortality ↓ (if given before ischemia)	
Abdominal aortic clamping in rats; 100 mg/kg i.v. before [37]	Attenuates lung injury	MDA in blood and lung ↓
Renal ischemia in rabbits; 15 mg/kg 24 hours and 1 hour before and 0.83 mg/minute during [34]	Ameliorates renal structure and function	PAF and PAF-like lipids ↓
		Myeloperoxidase activity ↓
Hepatic ischemia (clamping HA–PV) rats; 30, 100, 300, and 1,000 mg/kg 5 minutes before [38]	Bile flow and cholate secretion ↑	30 and 100 mg/kg:
	Extremely high dose is prooxidant	● AST and lipid peroxidation ↓
		● Prevents ↓ of cytochrome P450 1,000 mg/kg
		● Injury and loss of function ↑
IPC + hepatic ischemia (clamping left HA and PV) in rats; 100 mg/kg i.v. after IPC before clamping [41]	Ascorbate or IPC plus ascorbate after IPC reduce mitochondrial damage and dysfunction	Prevents mitochondrial:
		● Swelling
		● Peroxide ↑, MDA ↑
		● GSH and GSH/GSSG ↓
		● Glutamate dehydrogenase ↓
		● ATP ↓ (ascorbate plus IPC)
Liver ischemia in rats; 100 mg/kg i.v. 1 hour before [40]	Attenuates reperfusion liver injury	Attenuation of O_2_^–^ and NO release
Liver ischemia (clamping HA and PV) in rats; oral vitamin C for 5 days [39]	Attenuates myocardial injury and protects cardiac function after liver ischemia	Systemic hydroxyl radical ↓
		Myocardial MDA
Skeletal muscle ischemia in rats; oral vitamin C for 5 days [35]	Preserves muscle function	Muscle myeloperoxidase ↓
	Reduces edema	Neutrophil infiltration ↓
		Respiratory burst ↓
Skeletal muscle tourniquet in rats; 50 mg/kg i.v. before ischemia, before reperfusion, or both [36]	Preserves muscle function	Blood malondialdehyde↓
	Reduces edema	Muscle malondialdehyde =
		Neutrophil influx
**Sepsis**		
*Systemic and microcirculation*		
CLP in rats; 76 mg/kg i.v. directly after [32]	Restores blood pressure and density of perfused capillaries	
CLP in mice; 200 mg/kg 30 minutes before [6]	Improves microvascular constriction and arterial pressure responses to norepinephrine	iNOS expression ↓
		iNOS mRNA ↓
		ROS production ↓
CLP in mice; baseline and 23 hours after 200 mg/kg [48]	Restores arteriolar conducted vasoconstriction	Reduces increased:
		● nNOS activity
		● Nitrite/nitrate
CLP in rats; 76 mg/kg after 1 hour, 6 hours and 2 hours [44]	Prevents maldistributed blood flow and low arterial blood pressure	Blood flow impairment:
		● Requires NADPH oxidase
		● Reversal by ascorbate or BH_4_
		● eNOS dependent
FIP in mice; 10 or 200 mg/kg i.v. 6 hours after [47]	Prevents/reverses septic impairment of capillary blood flow for 18 hours and improves survival	Blood flow impairment depends on the NADPH oxidase subunit gp91phox
		Ascorbate effects are eNOS dependent
		Ascorbate suppresses iNOS ↑ activity
FIP in mice; 10 mg/kg i.v. prophylactic or delayed [46]	Prevention or reversal of septic platelet adhesion and/or flow stoppage	Capillary flow stoppage
		● eNOS dependent
		● Platelet adhesion predicts 90 %
CLP in mice; 200 mg/kg i.v. at baseline and 3 hours after [50]	Prevents vascular leakage	Inhibits production of:
		● O_2_^–^ and NO by NADPH oxidase, iNOS and nNOS
		● Peroxynitrite
		● 3-Nitrotyrosine-positive proteins ↓
		● Inhibits PP2A activation
		Preserves endothelial occludin phosphorylation
*Organ injury and function*		
Intraperitoneal LPS in guinea pigs; low vs. high vitamin C diet [51]	Dietary vitamin C	Hepatic lipid peroxidation ↓
	Increases hepatic vitamin C and vitamin E content	Hepatic protein carbonyls ↓
	Reduces oxidative damage to lipids and proteins	Hepatic GSH and GSH/GSSG ↑ (vitamin C + vitamin E)
CLP in rats; 100 mg/kg directly after [53]	Decreases hepatic injury	Suppresses AST and ALT ↑
	Improves drug-metabolizing function	Prevents GSH and GSH/GSSG ↓
		Prevents CYP1A1 and CYP2E1 mRNA, and CYP1A2 activity
FIP or LPS in mice; 200 mg/kg i.p. after LPS [54]	Attenuates sepsis-induced acute lung injury and improves 72-h survival	Preserves lung architecture and barrier
		Proinflammatory chemokine expression ↓ microvascular thrombosis ↓
		Nuclear factor-kappaB activation ↓
		Normalizes coagulation
*Immune defense against infection*		
*Klebsiella pneumonia* in mice; ascorbate deficient vs. ascorbate supplemented for 25 days [56]	Ascorbate deficiency increases death from infection	No effect on:
		● Cellular response
		● Amino acid and lipid peroxidation
		Higher concentration of bacteria in ascorbate deficiency

AA, ascorbic acid; ALT, alanine aminotransferase; AST, aminotransferase; BH_4_, tetrahydrobiopterin; CLP, cecal ligation and perforation; CPR, cardiopulmonary resuscitation; CYP, cytochrome P450; DHA, dehydroascorbic acid; eNOS, endothelial nitric oxide synthase; ES, electrical shock; FIP, feces injection into the peritoneum; GSH, reduced glutathion; GSSG, glutathione disulphide; HA, hepatic artery; IPC, ischemic preconditioning; LAD, left anterior descending; LPS, lipopolysaccharide; MDA, malondialdehyde (marker for lipid peroxidation); iNOS, inducible nitric oxide synthase; i.p., intraperitoneal; i.v., intravenous; NADPH, nicotinamide adenine dinucleotide phosphate; nNOS, neuronal nitric oxide synthase; NO, nitric oxide; O_2_^–^, superoxide; PAF, platelet-activating factor; PPA2, protein phosphatase 2A; PV, portal vein; ROS, reactive oxygen species; VF, ventricular fibrillation. ↑, increase; ↓, decrease; =, constant.

In a rat model of cardiac arrest (ventricular fibrillation and electrical shock), intravenous ascorbate at the start of resuscitation alleviated postcardiac arrest myocardial necrosis and mitochondrial damage, reduced lipid peroxidation and improved the resuscitation success rate and 72-hour survival [33]. In a pig model of coronary artery ischemia/reperfusion and preconditioning, intravenous ascorbate started 30 minutes before ischemic preconditioning or ischemia had no effect on infarct size, but abolished the reduction in infarct size by ischemic preconditioning [42]. In a rabbit renal ischemia model, ascorbate ameliorated renal structure and function [34]. Ascorbate also had a positive effect on muscle injury when administered intravenously (i.v.) during ischemia/reperfusion of the leg in rats [36]. Experiments with different intravenous ascorbate doses to rats prior to hepatic ischemia reperfusion found antioxidant effects at low and moderate doses and predominant pro-oxidant effects at extremely high doses (1,000 mg/kg) [38]. In addition, vitamin C administered after ischemic preconditioning but before clamping of the liver blood flow reduced hepatic mitochondrial damage and dysfunction [41]. Thus, in these animal models, ascorbate reduced ischemic organ injury and improved function, but abolished the protective effects of ischemic preconditioning on organ injury.

### Sepsis

The most frequently used sepsis animal models are feces injection into the peritoneum (FIP), cecal ligation and puncture (CLP) and intraperitoneal injection of LPS.

### Ascorbate depletion

Ascorbate plasma concentrations rapidly declined in lymphocytes and macrophages [43], muscle [6] and plasma [32,44] of septic rodents. Endotoxin also depleted myocardial ascorbate in guinea pig hearts after as early as 4 hours, even after dietary supplementation for 5 weeks [45].

### Systemic circulation, microcirculation and permeability

Ascorbate (76 mg/kg i.v.) restored blood pressure and prevented microvascular dysfunction in skeletal muscle of septic rats [32]. In a mouse model of FIP, ascorbate (10 mg/kg i.v.) inhibited impairment of microvascular perfusion when administered early (0 hours) and reversed septic platelet adhesion and flow impairment with late administration (after 6 hours) [46]. Similarly, ascorbate improved capillary blood flow by an eNOS-dependent mechanism when administered 6 hours after FIP [44,47]. In addition, ascorbate (200 mg/kg i.v.) administration before CLP protected against impaired arteriolar constriction and loss of catecholamine responsiveness and improved 24-hour survival in mice [6,48]. Ascorbate prevented arteriolar vasoconstriction by inhibiting eNOS uncoupling and iNOS-derived and neuronal nitric oxide synthase-derived NO production, when given before but also 3 hours after CLP in mice [48-50]. Moreover, ascorbate (200 mg/kg i.v.) prevented vascular leakage in a mouse CLP model by inhibiting excessive production of NO by iNOS and neuronal nitric oxide synthase, and of O_2_^–^ by NOX, and by inhibiting the activation of protein phosphatase 2A, thereby preserving occludin phosphorylation [50]. Ascorbate thus restored several seemingly contradictory disorders contributing the heterogeneity of the septic circulation. Ascorbate improved microcirculatory perfusion by NOX inhibition and arteriolar vasodilator responsiveness (neuronal nitric oxide synthase related), restored vasoconstrictor responsiveness by inhibiting iNOS expression and prevented vascular leakage.

### Effects on organ injury and function

Vitamin C prevented the increase in endotoxin-induced myocardial uric acid accumulation, a marker of ischemia-induced oxidative stress [45], and protected against endotoxin-induced oxidative damage to proteins in the guinea pig liver [51]. Ascorbate (100 mg/kg i.v.) reduced hepatic microvascular dysfunction during polymicrobial sepsis when administered immediately after CLP in rats by reducing oxidative stress and lipid peroxidation, and regulating hepatic vasoregulatory gene expression [52,53]. In addition, ascorbate prevented the sepsis-induced decrease in several cytochrome P450 enzyme activities, thereby improving drug-metabolizing function [53]. Ascorbate (200 mg/kg i.v.) also attenuated sepsis-induced acute lung injury in a mouse model of FIP or LPS [54,55] and improved 72-hour survival [54]. Finally, oral prefeeding with ascorbate decreased bacterial concentrations and improved survival after intraperitoneal injection of *Klebsiella pneumonia* in mice [56].

## Role of vitamin C in ischemia/reperfusion and sepsis: human volunteers

In human volunteers, both ischemia/reperfusion injury (20 minutes of forearm ischemia) [57] and low-dose LPS [58] reduced plasma vitamin C concentrations and diminished acetylcholine-induced, endothelial-dependent vasodilatation. High-dose (24 mg/minute) intra-arterial vitamin C increased BH_4_ concentrations [59], reduced neutrophil oxidative burst and completely restored the response to acetylcholine, but not to glyceryl-trinitrate (endothelium-independent dilatation), supporting its endothelial protective effect. Vitamin C also corrected the LPS-induced decreased responsiveness to norepinephrine and angiotensin II [60]. Both vasopressors act independent of the endothelium, but their effect is blunted by oxidative stress and inflammation. These volunteer studies originate from the same group.

Several preclinical *in vitro*, animal and volunteer studies thus show that vitamin C in moderate to high doses can reduce ROS-induced microcirculatory flow impairment, microvascular leakage, decreased responsiveness to vasoconstrictors, and myocardial and other organ injury. Contradictory results in ischemia/reperfusion can partially be explained by the timing of administration, because vitamin C abrogates ischemic preconditioning. Furthermore, vitamin C reduces the overwhelming neutrophil response and inhibits bacterial replication.

## Plasma concentrations, dose and pharmacokinetics of vitamin C: patients

### Plasma concentrations

Vitamin C plasma concentrations depend on absorption, the distribution volume, cellular uptake, consumption and renal reabsorption and excretion. Patients with sepsis, hemorrhage, multiple organ failure, stroke, traumatic brain injury or after cardiac surgery have low vitamin C concentrations in plasma [61-68] and leukocytes [69], probably due to increased consumption in the cell [70] and high leukocyte turnover. Since intracellular ascorbate concentrations in mononuclear leucocytes and in granulocytes are respectively 80 and 25 times higher than in plasma [71], a high production and turnover of these cells contributes to depletion. Low plasma concentrations correlate with inflammation (C-reactive protein) [64] and multiple organ failure [55], suggesting consumption during oxidative stress.

### Dosing

Recommended doses of vitamins are generally based on preventing deficiency in healthy humans. In healthy volunteers, manifestations of vitamin C deficiency (fatigue and/or irritability) occurred at plasma concentrations below 20 μmol/l. Clear scurvy can develop below 11 μmol/l [13]. With sufficient vitamin C intake (100 to 300 mg/day), plasma concentrations plateau at 70 to 85 μmol/l and do not exceed 220 μmol/l at maximal oral intake (3 g six times daily) [72]. Oral dose is limited by intestinal absorption and high oral intake causes diarrhea [73]. Urinary excretion depends on the plasma concentration and is minimal at low plasma concentrations due to active tubular reabsorption [74,75]. Threshold plasma concentration for excretion may be 55 μmol/l [72].

Ascorbate is transported into the cell by ascorbate-specific membrane transporters and less so as dehydroascorbic acid via glucose transporters [76]. Within the cell, dehydroascorbic acid can be rapidly reduced to ascorbate, thereby recycling ascorbate [77]. The ascorbate transporter SVCT1 is expressed predominantly in epithelial tissues such as the intestine and kidney, maintaining optimal vitamin C concentrations in the body. The ascorbate transporter SVCT2 delivers ascorbic acid to tissues [78]. Immune and inflammatory cells have intracellular concentrations 10 to 80 times higher than plasma, protecting them against ROS generated by respiratory burst or phagocytosis [71]. Neurons have concentrations as high as 10 mmol/l, sufficient for scavenging O_2_^–^ [79,80]. Of note, ascorbate does not pass the blood–brain barrier. However, dehydroascorbic acid does so via the glucose transporter GLUT1 and is reduced to ascorbate after uptake in neurons. Intravenous administration of dehydroascorbic acid confers supraphysiologic concentrations of ascorbate in the brain [81].

Notably, concentrations attained with high oral dosing are sufficient to modulate enzymes such as nicotinamide adenine dinucleotide phosphate but not for scavenging O_2_^–^, which reacts with NO at a rate 10^5^-fold greater than that with ascorbate [21]. A plasma concentration of 10 mmol/l would be required to compete with NO and for complete restoration of NO bioavailability. High plasma concentrations can be obtained with intravenous administration. However, mild dietary supplementation of vitamin C reduced peroxynitrite formation and atrial electrophysiological remodeling induced by rapid pacing in dogs [82], probably due to higher intracellular vitamin C concentration.

High intravenous vitamin C doses, up to 3 to 6 g daily, are needed to restore normal plasma concentrations in critically ill patients [83]. To attain plasma concentrations over 10 mmol/l for 3 hours, a short-term infusion of 30 to 100 g would be required [73,74]. High pharmacological doses of vitamin C seem to be well tolerated [84,85]. Prolonged oral intake of high-dose vitamin C increases the risk of oxalate kidney stones [86]. However, this complication has not been reported with short-term high intravenous dosing [87]. Of note, low dose ascorbate can also act as a pro-oxidant [37]. However, after a 1 g vitamin C intravenous infusion, ascorbyl radical concentrations increased much more in healthy controls than in septic patients, who had a lower baseline concentration [88].

## Role of vitamin C in ischemia/reperfusion: clinical studies

Whereas most preclinical studies investigate the role of vitamin C alone, clinical studies often used a combination of antioxidants. Some studies are performed in conditions similar to animal models, reporting the use of vitamin C before the ischemic incident (coronary bypass surgery) or directly at reperfusion (percutaneous coronary interventions) or shock resuscitation (burns). In the critically ill studies, combinations of antioxidants were generally given after the hyperacute phase.

### Percutaneous coronary intervention

In patients undergoing elective percutaneous coronary intervention, vitamin C in a dose of 1 g over 1 hour improved microcirculatory reperfusion, and left ventricular and renal function [89]. This improvement was associated with reduced markers of oxidative injury.

### Cardiac surgery

Atrial fibrillation is the most common arrhythmia after cardiac surgery, developing in 15 to 50 % of patients depending on several risk factors [90]. Atrial fibrillation increases short-term and long-term morbidity and length of hospital stay [90,91], and necessitates anticoagulation to prevent stroke*.* Its pathogenesis is multimodal but accumulating evidence indicates a role of oxidative stress [92-94]. Ischemia/reperfusion, atrial stress and angiotensin increase atrial NOX activity, which is associated with postoperative atrial fibrillation [94-96]. Oxidative damage initiates breakdown of cell membranes, mitochondrial dysfunction, calcium overload, apoptosis and also inflammation by signaling activation of nuclear factor-kappaB and activator protein-1 transcription factors [97], thereby initiating electrophysiological remodeling.

After cardiac surgery, a massive depletion of vitamin C has been observed [66]. Several studies suggest a beneficial effect of vitamin C on the occurrence of new atrial fibrillation and some on enhanced recovery, although not all studies are positive (Table 2). In a matched control study, the perioperative use of vitamin C reduced the incidence of new postoperative atrial fibrillation in patients undergoing coronary artery bypass grafting [82]. A subsequent randomized controlled trial found a reduction in postoperative atrial fibrillation when adding vitamin C to a β-blocker [98]. In the largest trial, vitamin C did not reduce atrial fibrillation, but it reduced time on mechanical ventilation [99]. Another clinical trial found a reduction in atrial fibrillation, but its incidence in the control group was extremely high [100]. The most recent randomized controlled trial found a reduction in postoperative atrial fibrillation comparing preoperative ω-3 poly-unsaturated fatty acids with vitamin C and vitamin E supplementation with placebo (see Table 2) [95]. An older Chinese study using a very high dose of intravenous vitamin C (250 mg/kg) found less cardiac injury, better cardiac performance and shorter intensive care and hospital stay [101].

**Table 2:** **Controlled studies on the effect of vitamin C in cardiac surgery patients**

**Study**	**Design**	**Intervention**	**Number of patients**	**Incidence of new POAF (%)**	***P*** **value**	**Other clinical benefits**
Dingchao and colleagues [101]	Controlled; patients undergoing cardiopulmonary bypass	i.v. vitamin C; 250 mg/kg i.v. before	45			MDA ↓; CK, CK-MB ↓; postbypass defibrillation 0 vs. 12.5 %; CI ↑, LOS ICU ↓, LOS hospital ↓
		Control	40			
Carnes and colleagues [82]	Matched control; CABG	Oral vitamin C; 2 g night before, 500 mg daily for 5 days	43	16.3	0.048	
		Matched control	43	34.9		
Eslami and colleagues [98]	RCT; CABG	β-Blocker + oral vitamin C; 2 g night before, 1 g twice daily for 5 days	50	4	0.002	
		β-Blocker alone	50	26		
Bjordahl and colleagues [99]	RCT; CABG	Oral vitamin C; 2 g night before, 1 g twice daily for 5 days	89	30.3	0.985	Shorter time on ventilator, 1.2 vs. 1.4 days, *P* = 0.032
		Placebo	96	30.2		
Papoulidis and colleagues [100]	RCT; CABG	i.v. vitamin C; 2 g 3 hours before CPB	85	44.7	0.041	Time to SR conversion ↓, LOS hospital ↓, LOS ICU ↓
		i.v. saline	85	61.2		
Rodrigo and colleagues [95]	RCT	Preoperative PUFA; 2 g/day for 5 days; vitamin C 1 g/day + vitamin E 400 IU/day for 2 days preoperatively and postoperatively until discharge	103	9.7	<0.001	Oxidative stress-related biomarkers in atrial tissue ↓
		Placebo^a^	100	32		

CABG, coronary artery bypass surgery; CI, cardiac index; CK, creatinine phophokinase; CK-MB, creatinine phosphokinase muscle, brain isoenzyme; CPB, cardiopulmonary bypass; i.v., intravenously; LOS, length of stay; MDA, malondialdehyde; POAF, postoperative atrial fibrillation; PUFA, ω-3 polu-unsaturated fatty acids containing eicosapentaenoic and docosahexaenoic acids in a 1:2 ratio; RCT, randomized controlled trial; SR, sinus rhythm; ↑, increase; ↓, decrease; =, constant. ^a^Placebo contained 500 mg inert microgranules, 825 mg triglycerides and 500 mg vegetable oil per capsule.

Studies differ in the timing, route and dose of vitamin C, and in the combination of other antioxidants. Timing may be crucial in cardiac surgery, because preoperative episodes of ischemia and reperfusion protect the myocardium against perioperative ischemic damage and vitamin C may hamper the beneficial effects of ischemic preconditioning on reducing infarct size [102].

### Critically ill patients

Several clinical trials in critically ill patients have reported favorable results of high-dose vitamin C alone [90,91], or in combination with vitamin E [103,104] or with selenium, zinc and vitamin B [105,106] (Table 3). The main beneficial outcomes include reduction in pulmonary morbidity and new organ failure, less mechanical ventilation days and shorter length of ICU and/or hospital stay. Some studies measured lower markers of oxidative stress [84,107]. Although ROS can signal host defense in low concentrations, the parallel finding of less oxidant stress and less organ dysfunction suggests a beneficial effect of reducing overwhelming ROS during critical illness. The largest study, however, using a mixture of micronutrients including oral vitamin C, found no effect on 28-day mortality or length of stay. Of note, the control group in Berger and colleagues’ study received 500 mg/day vitamin C [105].

**Table 3:** **Controlled studies on the effect of vitamin C in critically ill patients**

**Study**	**Design**	**Intervention**	**Number of patients**	**Outcome**
Nathens and colleagues [104]	RCT; trauma and MOF	i.v. vitamin C 1 g three times daily; enteral vitamin E 1,000 IU three times daily	301	Pulmonary morbidity ↓, new MOF ↓, LOS ventilation ↓, LOS ICU ↓
		With TPN, vitamin C 100 mg and vitamin E 10 IU daily; with EN, vitamin C 340 mg/l, vitamin E 60 IU/l	294	
Crimi and colleagues [107]	RCT; critically ill (mainly trauma, cardiogenic shock)	Vitamin C 500 mg/day and vitamin E (400 IU/day) in EN	105	Ventilator-free days ↓, 28-day mortality ↓
		Saline solution for 10 days	111	
Collier and colleagues [103]	Prospective vs. retrospective 1-year cohort; trauma	i.v. or oral vitamin C 1 g three times daily + oral vitamin E 1,000 IU three times daily + selenium 200 μg i.v.	2,272	LOS ICU ↓, LOS hospital ↓, mortality ↓; OR 0.32, 95 % CI 0.22 to 0.46
		Standard therapy	2,022	
Berger and colleagues [105]	RCT; complicated cardiac surgery, trauma, SAB	Selenium 540 i.v. day 1, 270 μg days 2 to 5; zinc 60 mg i.v. day 1, 30 mg days 2 to 5; vitamin B1 305 mg i.v. day 1, 205 mg days 2 to 5; vitamin C 2.7 g i.v. day 1, 1.6 g days 2 to 5; vitamin E 600 mg i.v. day 1, 300 mg days 2 to 5	102	New organ failure ND, new infections ND, LOS shorter in trauma, CRP ↓ in cardiac surgery and trauma, recovery of health after discharge ↑
		Vitamin B1 100 mg i.v. days 1 to 3 (both groups); vitamin C 500 mg i.v. days 1 to 5 (both groups)	98	
Heyland and colleagues [106]	RCT, 2 × 2 factorial; critically ill adults with multiple organ failure	Selenium 500 μg i.v., selenium 300 μg or zinc 20 mg or β-carotene 10 mg; vitamin E 500 mg or vitamin C 1,500 mg	307	No difference in 28-day mortality or length of stay
**Burn**		Placebo	300	
Tanaka and colleagues [84]	RCT; severe burn <2 hours	Ringer lactate + 66 mg/kg/hour vitamin C	19	Fluid requirements ↓, body weight gain ↓, PF ratio ↑, days on mechanical ventilation ↓
		Ringer lactate for 24 hours	18	
Kahn and colleagues [85]	Retrospective; severe burn <10 hours	Ringer lacate + 66 mg/kg/hour vitamin C	17	Fluid requirements ↓, urinary output ↑
		Ringer lactate for 24 hours	16	

CI, confidence interval; CRP, C-reactive protein; EN, enteral nutrition; i.v., intravenously; LOS, length of stay; OR, odds ratio; MOF, multiple organ failure; ND, no difference; PF, ratio of partial oxygen pressure in arterial blood to fraction of inspired oxygen; RCT, randomized controlled trial; TPN, total parenteral nutrition; SAB, subarachnoid bleeding. ↑, increase; ↓, decrease; =, constant.

Combined administration with vitamin E and other micronutrients obscures the role of vitamin C. However, vitamin C regenerates vitamin E, and vitamin E is only consumed after depletion of vitamin C [108]. Two small studies in burn patients studied a very high dose of vitamin C alone (66 mg/kg/hour) for about 24 hours and found a reduction in resuscitation volume, better gas exchange and less days on mechanical ventilation [84] and increased urinary output [85], probably indicating less capillary leak. No signs of acidosis or renal insufficiency were found with this high dose. However, although vitamin C reduced morbidity in some studies, a mortality reduction was not found. We hypothesize that the effect of vitamin C can be improved by very early administration of a high intravenous dose as part of the resuscitation bundle in patients with shock.

## Conclusion

This narrative review summarizes the role of vitamin C in mitigating ROS-induced microcirculatory impairment and associated organ failure in ischemia/reperfusion or sepsis. Preclinical studies show that high-dose vitamin C can prevent or restore ROS-induced microcirculatory flow impairment, prevent or restore vascular responsiveness to vasoconstrictors, preserve endothelial barrier and augment antibacterial defense. These protective effects against oxidative stress seem to mitigate organ injury and dysfunction, and promote recovery in most but not all clinical studies after cardiac revascularization and in critically ill patients.

Of note, many questions remain to be solved, including the optimal dose, timing, combination of vitamin C with other antioxidants and the inhibiting effect of vitamin C on the protection of ischemic preconditioning. However, high-dose vitamin C provides a cheap, strong and multifaceted antioxidant. Future research should answer the question of whether short-term high-dose intravenous vitamin C can mitigate the overwhelming oxidant cascade and thereby improve resuscitation of the macrocirculation and microcirculation and limit cellular injury in critically ill patients.

## Abbreviations

BH_4_: Tetrahydrobiopterin
CLP: Cecal ligation and puncture
eNO: Endothelial nitric oxide
eNOS: Endothelial nitric oxide synthase
FIP: Feces injection into the peritoneum
i.v.: Intravenously
iNOS: Inducible nitric oxide synthase
LPS: Lipopolysaccharide
NO: Nitric oxide
NOX: Nicotinamide adenine dinucleotide phosphate-oxidase
O_2_^–^: Superoxide
ROS: reactive oxygen species
